# Dendritic epidermal T cells secreting exosomes promote the proliferation of epidermal stem cells to enhance wound re-epithelialization

**DOI:** 10.1186/s13287-022-02783-6

**Published:** 2022-03-21

**Authors:** Mian Liu, Zhihui Liu, Yunxia Chen, Shiya Peng, Jiacai Yang, Cheng Chen, Jue Wang, Ruoyu Shang, Yuanyang Tang, Yong Huang, Xiaorong Zhang, Xiaohong Hu, Yih-Cherng Liou, Gaoxing Luo, Weifeng He

**Affiliations:** 1 State Key Laboratory of Trauma, Burn and Combined Injury, Institute of Burn Research, Southwest Hospital, Third Military Medical University (Army Medical University), Chongqing, 400038 China; 2 Chongqing Key Laboratory for Disease Proteomics, Chongqing, 400038 China; 3 Department of Dermatology, Xinqiao Hospital, Army Military Medical University, Chongqing, 400038 China; 4 Academy of Biological Engineering, Chongqing University, Chongqing, 400038 China; 5 Department Biological Sciences, National University of Singapore, Singapore, 117543 Singapore

**Keywords:** Epidermal stem cells, Proliferation, Re-epithelialization, Dendritic epidermal T cells, Exosomes

## Abstract

**Background:**

Efficient re-epithelialization is important for successful skin wound healing. The proportion of epidermal stem cells (EpSCs) and dendritic epidermal T cells (DETCs) determines the extent of wound re-epithelialization, especially in large areas of skin tissue loss. However, it remains unknown whether and how DETCs regulate the status of EpSCs to impact wound re-epithelialization.

**Methods:**

To investigate how DETCs regulate EpSCs in skin re-epithelialization, we utilized normal or full-thickness skin deficient wide type (WT) mice and Tcrσ knockout (Tcrσ^−/−^) mice with DETCs or DETCs-derived exosomes (Exos) treatment. Flow cytometry analysis (FCAS), BrdU labelled experiments, immunofluorescence and immunohistochemical assays were performed to detect the proportion of EpSCs in the epidermis. Wound closure rate and re-epithelialization were assayed by a macroscopical view and hematoxylin–eosin (H&E) staining. EpSCs in vitro were co-cultured with DETCs in a transwell-dependent or -independent manner, or supplement with GW4869 or Exos (5 µg/mL, 15 µg/mL and 45 µg/mL), and the proliferation of EpSCs was detected by means of FCAS and CFSE.

**Results:**

Our data showed that the proportion of CD49f^bri^CD71^dim^ cells, K15^+^ cells and BrdU^+^ cells in the normal epidermis of Tcrδ^−/−^ mice had no significant difference compared to WT mice. For wounded Tcrδ^−/−^ mice, DETCs treatment increase the proportion of CD49f^bri^CD71^dim^ cells, K15^+^ cells and BrdU^+^ cells in the epidermis around the wound in comparison to PBS treatment. DETCs significantly increased the number of CD49f^bri^CD7^dim^ cells and K15^+^ cells through transwell-dependent or -independent manners relative to control group. Furthermore, Exos stimuli remarkedly promote the proliferation of EpSCs compared to control group, while the increasement was suppressed when DETCs were interfered with GW4869. Gross observation and H&E staining showed that Exos significantly accelerated wound closure and increased re-epithelialization length in Tcrδ^−/−^ mice when compared to control mice. Additionally, we found in vivo that Exos observably facilitated the proliferation of CD49f^bri^CD7^dim^ cells and K15^+^ cells.

**Conclusions:**

We revealed that DETCs enhanced the proliferation of EpSCs in the epidermis around the wounds to accelerate re-epithelialization in which Exos played important roles in the remote regulation of EpSCs proliferation. Together, these findings suggest a mechanistic link among DETC-derived exosomes, the proliferation of EpSCs, and wound re-epithelialization in the skin.

## Introduction

Efficient re-epithelialization is required for successful skin wound healing [1, 2]. The proliferative potential of epidermal tissue determines the effectiveness of wound re-epithelialization, especially in large areas of skin tissue loss [3–5]. The proportion and proliferation of epidermal stem cells (EpSCs) are one of two key factors affecting the regenerative potential of the epidermis around wounds after skin injury [6, 7]. Traditionally, epidermal cells with CD49f^bri^CD71^dim^ or K15^+^ phenotypes have been identified as EpSCs [8–10]. EpSCs commonly present a resting status relative to other cells, maintaining skin cell homeostasis through self-renewal and keratinocyte renewal in the normal epidermis [9]. Yet, when the skin is damaged, EpSCs around the area of wound edge are activated to promote wound healing by several mechanisms including proliferation, migration and differentiation, which are the cellular basis of the wound re-epithelialization process [11].

γδ T cells, which are mainly distributed in the skin, lung and intestine, are involved in the recognition of restricted antigens of the nonmajor histocompatibility complex and thus become important in the immune response [12–17]. Dendritic epidermal T cells (DETCs), the main γδ T cells resident in the mouse epidermis, extend dendritic structures from the surface of their cell bodies, which constantly interact with mature keratinocytes in the outer layer of the epidermis as well as immature keratinocytes in the basal layer [18]. DETCs play an irreplaceable role in wound closure by providing a great source of insulin-like growth factor (IGF)-1 and keratinocyte growth factor (KGF)-1/2, which contribute to rapid wound re-epithelialization [19]. In contrast, the lack of DETCs at the area of wound edge leads to a lower proliferative activity of epidermal cells and higher EpSC apoptosis in the epidermis for the cutaneous wounds, resulting in delayed wound repair [20]. As mentioned above, EpSCs and DETCs in the epidermis both facilitate re-epithelialization of skin wounds. However, the precise role of how DETCs regulate the cellular functions of EpSCs to enhance re-epithelialization remains still unknown.

Exosomes, an extracellular vesicle with 30–150 nm in diameter, are secreted by multiple types of cells and rich in specific proteins, lipids, and nucleic acids [21–23]. Exosomes have been shown to participate in intercellular communication by transferring signals or molecules to other cells, which play an essential role in human health and disease [24]. For instance, a study performed by He et al. showed that mesenchymal stem cells (MSCs) induced macrophage toward M2 polarization and accelerate wound healing by transferring exosome-derived microRNA targeting pknox1 [25]. Moreover, a similar study on the role of exosomes in the progression of diabetic foot ulcer healing employed exosomes derived from adipose stem cells overexpression nuclear factor-E2-related factor 2 (Nrf2) (Nrf2-ADSC-Exos) and revealed that Nrf2-ADSC-Exos significantly increased the granulation tissue formation, angiogenesis, and the levels of growth factor expression as well as reduced levels of inflammation and oxidative stress-related proteins in wound beds in comparison to ADSC-Exos [26]. Although exosomes are more capable of signal transduction and transport than free cytokines, there is no evidence on whether DETCs function by exosome exocytosis. Hence, the underlying mechanism that DETCs interact with EpSCs in the course of wound healing need to be clarified.

In the present study, in order to investigate how DETCs regulate the proliferation of EpSCs promoting the re-epithelization, the full-thickness skin excision models of wide type (WT) and Tcrδ knockout (Tcrδ-/-) mice are created. Firstly, 5-bromo-2-deoxyuridine (BrdU) reagent was intraperitoneally injected to label EpSCs in vivo to access the effects of DETCs on the proliferation of EpSCs. Moreover, the exosomes derived from DETCs (Exos) were utilized to evaluate the proliferation of EpSCs and cutaneous wound re-epithelization. Taken together, our data revealed a novel mechanism by which Exos can promote the proliferation of EpSCs in the epidermis around wounds and accelerated wound re-epithelialization, which might provide a potential treatment strategy for hard-healing wounds or ulceration.

## Materials and methods

### Animals

All C57BL/6 (WT) mice used in these experiments were purchased from the Experimental Animal Department of Army Medical University (Third Military Medical University) with animal license number: SYXK20170002. Tcrδ knockout (Tcrδ^−/−^) mice on a C57BL/6 background were obtained from Jackson Laboratory. All experiments were performed under conventional animal raising conditions in conformity with ethical guidelines and approved by the Animal Ethics Committee of Army Military Medical University.

### Preparation and treatment of excision wounds

Wounds were generated on age- and sex-matched WT mice or Tcrδ^−/−^ mice (8–10 weeks, male, *n* = 6/group). After anesthesia, the dorsal surface of each mouse was shaved, and a full-thickness excision wound was made on both dorsal skin of the mouse using a sterile punch with a diameter of approximately 6 mm. DETCs (1 × 10^5^/wound in 50µL PBS) or control vehicle (50µL PBS) was subcutaneously injected into the wound bed immediately after wounding. For DETCs-derived exosomes (Exos) administration experiments, Exos (15 µg/wound in PBS) or equal volume PBS was subcutaneously injected into the area of wound edge once a day for 8 days. The size of the wound area was recorded daily by a digital camera to estimate the wound healing process at each time point. The area of wound healing was measured using the ImageJ Pro-Plus software (NIH, USA), and the wound-healing percentage was calculated using the following formula:

$${\text{Wound - healing rate}}\,\left( \% \right) = \frac{{{\text{AW}}_{i} - {\text{AW}}_{n} }}{{{\text{AW}}_{i} }} \times 100\%$$

where AW_*i*_ represents the area of the initial wound (the actual size after wound creation), and AW_*n*_ represents the area of the wound at different time points post-injury. All wounds were uncovered, and mice were individually assigned into sterile bedding.

### Isolation of epidermal tissues

After depilation, the dorsal skin of WT and Tcrδ^−/−^ mice was collected and cut into 1.0 × 1.0 cm pieces. The skin specimens were washed twice using PBS and incubated with 5 mg/mL Dispase II solution (Sigma) for 6 h at 4 °C to separate the epidermis and dermis. The epidermal sheets were immersed in 0.25% Trypsin solution for 20 min at 37 °C, washed with RPMI medium (Gibco) containing 10% fetal bovine serum (FBS) (Gibco) and filtered through a 70-µm sterile strainer (NEST). For experiments involving the wounded epidermis, skin less than 5 mm away from the wound edge was collected to obtain epidermal cells around the wound area.

### Identification of EpSCs

For CD49f-CD71 staining, an epidermal single-cell suspension was generated from the back skin of WT and Tcrδ^−/−^ mice. Cells were first blocked with anti-CD16/32 monoclonal antibodies (eBioscience, Species: mouse, Isotype: IgG1, κ, Clone: 3G8) for 20 min and then incubated separately with an anti-human/mouse CD49f antibody (eBioscience, Species: Rat, Isotype: Rat IgG2 a, κ, Clone: GoH3) and an anti-mouse CD71/transferrin receptor antibody (BD Biosciences, Species: Rat, Isotype: Rat IgG1, κ, Clone: C2) in phosphate buffer solution (PBS) for 30 min at 4 °C.

For intracellular K15 staining, EpSCs were resuspended in the cell fixation permeabilization solution (Invitrogen) and incubated in the dark for 30 min at room temperature. The cells were washed with 1 × permeabilization buffer (Invitrogen) and incubated with an anti-mouse K15 antibody (Abcam) as 1:100 (K15 antibody:1 × permeabilization buffer) ration in the dark for 2 h at room temperature. Furthermore, the cells were stained with a BV421-conjugated secondary antibody (Biolegend) in the dark in the 1 × permeabilization buffer for 30 min at room temperature. The stained EpSCs were then detected using an Attune Acoustic Focusing Cytometer (Life Technologies), and the data were analyzed by a FlowJo software (Tree Star Incorporation).

### Immunofluorescence

The epidermal tissues of WT and Tcrδ^−/−^ mice were obtained, fixed, and created into 5-µm frozen sections at − 20℃. The specimens were then washed twice with 1 × PBS at room temperature. Then, the sections were permeabilized using 0.3% Triton X-100 solution for 15 min at room temperature and washed again with 1 × PBS. The samples were incubated with 3% BSA solution for 30 min at 37 °C. For staining, an AF594-conjugated anti-mouse CD3 antibody (Biolegend, Species: Rabbit, Isotype: IgG2b, κ, Clone: 17A2), AF647-conjugated anti-mouse K14 antibody (Abcam, Species: Rat, Isotype: IgG, Clone: EPR17350) and anti-mouse K15 primary antibody (Abcam, Species: Rabbit, Isotype: IgG, Clone: EPR1614Y) were incubated with samples overnight at 4 °C, and then with AF488-conjugated secondary antibodies (Abcam) targeting the K15 primary antibody for 30 min at 37 °C. Finally, the specimens were observed under a Zeiss fluorescence microscope. The expression levels of K15 were quantified by calculating average optical density (AOD) of positive signal using the Image J Pro-Plus software (NIH, USA). The data of WT group were normalized for analyzing the relative expression levels of Tcrδ^−/−^ groups.

### Immunohistochemistry

Based on previous research [27], 5-bromo-2-deoxyuridine (BrdU) was used to label EpSCs in vivo. First, newborn WT or Tcrδ^−/−^ mice on day 3 after birth were intraperitoneally injected with 50 mg/kg BrdU (Sigma) twice a day (8 a.m. and 7 p.m.) for 3 days. After seven weeks, epidermal cells retaining the label were identified as EpSCs.

To detect positive BrdU (BrdU^+^) EpSCs, these skin specimens were immobilized, embedded and created into 8-µm paraffin sections. The sections of these mice were deparaffinized in xylene and hydrated in a series of ethanol solutions. The sections were treated with citrate buffer (pH 6.0) at 96 °C for 20 min and incubated with an anti-BrdU antibody (Abcam, 1:200) overnight at 4 °C. The samples were washed and incubated with a corresponding secondary antibody (1:400) (Abcam) for 30 min at 37 °C. The sections were incubated with diaminobenzidine solution (Solarbio) and counterstained with hematoxylin (Solarbio). The BrdU-labeled EpSCs were observed under a BX51 microscope (Olympus). The expression levels of BrdU were quantified by calculating average optical density (AOD) by the Image J Pro-Plus software (NIH, USA). The data of WT group were normalized for analyzing the relative expression levels of Tcrδ^−/−^ groups.

### Isolation and culture of DETCs

Briefly, the epidermal tissues from 8- to 12-week-old male C57BL/6 WT mice were separated and obtained with 5 mg/mL dispase II solution. The specimens were created into a single-cell suspension using a 0.3% trypsin/GNK solution and filtered through a 70-µm sterile strainer. Then, lympholyte-M (Cedarlane Laboratories) was added into the single-cell suspension to enrich DETCs. For cellular culture, the cell density was adjusted to 1–2 × 10^6^/mL, and the cells were seeded into 48-well plates. The DETCs were cultured using RPMI medium with 10% exosomes-deleted FBS (Umibio), 1 mM sodium pyruvate (Sigma Aldrich), 10 ng/mL mouse rIL-2, 25 mM HEPES, 100 U of penicillin, 50 µM 2-ME (Biosharp), 2 mM glutamine, 100 M nonessential amino acids (Gibco), 100 µg of streptomycin and 1 µg/mL Concanavalin A (Sigma Aldrich) according to previously described protocol [28]. DETCs were incubated under 5% CO_2_ at 37 °C for four weeks to improve the purity of DETCs to > 95%, which were used for experiments in next four weeks.

### Isolation and culture of EpSCs

The full-thickness skin of newborn WT mice was obtained and incubated with 0.5% Dispase II solution for 6 h at 4 °C to separate the epidermis and dermis. To prepare epidermal single-cell suspensions, the epidermal samples were immersed in 0.3% trypsin solution for 15 min at 37 °C, washed with RPMI medium (containing 10% FBS) and filtered through a 70-µm sterile strainer. For EpSC culture, the filtered cells were centrifuged at 250 × g for harvesting epidermis cells. The pellet was resuspended and cultured with complete medium containing K‐SFM (Gibco), bovine pituitary extract (20‐30 mg/mL), cholera toxin (1 × 10^−10^ mol/L; Sigma), mouse recombinant epidermal growth factor (0.1‐0.2 ng/mL; Peprotech), mouse epidermal growth factor (10 ng/mL; BD), streptomycin/penicillin solution (100 IU/L; Gibco) and calcium chloride (0.05 mmol/L). The medium was changed every 48 h. The cells at 2–4 passage were used for identification as well as other experiments.

### Coculture of DETCs and EpSCs

DETCs were seeded into a 48-well plate and cocultured with EpSCs at an EpSCs:DETCs ratio of 10:1 for 72 h in EpSC culture medium (referred to as the DETCs group). In the noncontact Transwell coculture system, DETCs were treated in the absence or presence of 1 µM GW4869, a neutral sphingomyelinase inhibitor, and seeded into the upper compartment with a 0.4-μm pore size (Corning), while EpSCs were plated in the lower compartment (referred to as the DETCs-Transwell group). The control group (only EpSCs) was treated with the same volume of DETC medium.

### Proliferation assay

A carboxyfluorescein diacetate succinimidyl ester (CFSE) dye (Thermo Fisher Scientific) was used as a fluorescence tracking approach to detect the EpSC proliferation [29]. Briefly, EpSCs were labeled with a 5 μM CFSE dye solution for 20 min at 37 °C in the dark. Then, the cells were washed with RPMI medium containing 10% FBS to remove residual dye. The labeled EpSCs were treated for 3 days with DETCs (EpSCs:DETCs = 10:1) or DETCs-derived exosomes (0, 5, 15, 45 μg/mL). To determine cell proliferation, EpSCs were harvested and detected by flow cytometry. The expression levels of CFSE were analyzed by calculating the mean fluorescence intensity (MFI) values using a FlowJo software. The greater MFI values indicated the lower proliferation rate.

### Isolation of DETCs-derived exosomes

Briefly, DETCs were incubated with DETC culture medium with 10% exosome-free FBS (Umibio) for 48 h. The cell supernatant was collected, filtered through a 0.22 μm sterile strainer (NEST) and then concentrated 10 times by filtration using a 50 kDa ultrafiltration tube (Merck). The ExoQuick reagent (System Biosciences) was added into the concentrated supernatants and incubated for not less than 12 h at 4 °C according to manufacturer's instructions [30]. The mixed solution was centrifuged at 3000 × g for 30 min to isolate exosomes. The obtained pellet was resuspended in PBS for experiments.

### Western blot

Briefly, DETCs-derived exosomes and 3T3 cells were lysed in a RIPA lysis buffer (Beyotime) supplementing with a protease inhibitor and PMSF reagent (Beyotime) for 30 min on ice. The extracted protein was quantified by a BCA protein assay kit (Thermo Fisher Scientific). Then, a total of 30 µg protein samples were separated by 10% SDS-PAGE and transferred to polyvinylidene difluoride (PVDF) membranes. The PVDF membranes were then incubated with primary antibodies overnight at 4 °C using anti-mouse Calnexin (1:1000, ab133615, Abcam), anti-mouse CD63 (1:1000, ab217345, Abcam) or anti-mouse TSG101 (1:1000, ab125011, Abcam). Subsequently, the membranes were incubated with HRP-labeled secondary antibodies (Sungene Biotech) for 60 min at room temperature. Chemiluminescence reagents and the ChemiDoc™ XRS western blot detection system (Bio-Rad) were used to detect the proteins of interest.

### Characterization of DETCs-derived exosomes

The concentration of exosome suspension was adjusted to 1–1.5 µg/mL, and dropped onto the copper mesh, baked for 30 min at 65 °C, and then labeled with phosphotungstic acid. The transmission electron microscopy (TEM-1400Plus) was utilized to observe the morphology of Exos. Moreover, the size of Exos was also detected by a Zetasizer Nano ZSP (Malvern).

## Results

### DETCs have no significant effects on the proliferation of EpSCs in the normal epidermis

**Fig. 1:**
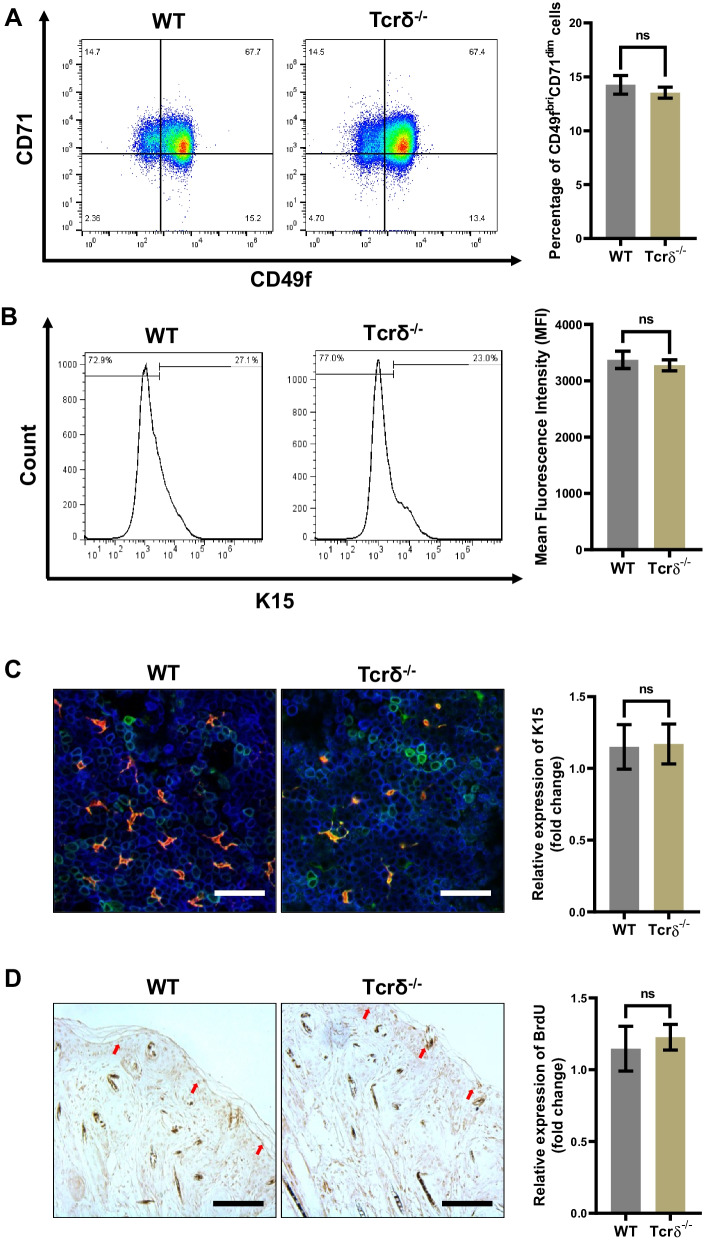
The proportion of EpSCs in the normal epidermis was unaffected by DETCs deficiency. **A**–**C** Normal epidermis was isolated from age- and sex-matched WT and Tcrδ^−/−^ mice. The proportions of CD49f^bri^CD71^dim^ cells (**A**) and K15^+^ cells (**B**) were analyzed by FACS. **C** The morphology (left panel) and number per visual field (right panel) of K15^+^ cells (green) were analyzed by means of immunofluorescence (IF). K14^+^ cells (blue) and epidermal T cells (red) are also shown in the images. Scale bar: 100 µm. **D** Paraffin sections of skin tissues from 8-week-old WT and Tcrδ^−/−^ mice that had been labeled with BrdU after birth. BrdU^+^ cells in these tissues were detected by means of immunohistochemistry (IHC). Scale bar: 100 µm. The arrow indicates positively stained cells (tan staining). All data are representative of at least three independent experiments and represent mean ± SD of indicated number of mice per group. The *p* value was calculated by Student’s unpaired *t* test (**A**–**C**) (^ns^*p* > 0.05)

Homeostasis of the epidermal barrier of the skin is maintained by the division and differentiation of EpSCs in the basal layer and the gradual migration of these cells to the outermost epidermis [11]. Concurrently, DETCs participate in maintaining the integrity of the skin epidermis by regulating the behavior of epidermal cells. To further investigate the impacts of DETCs on the proliferation of EpSCs in normal skin, we examined the proportions of EpSCs in normal epidermis isolated from age- and sex-matched WT and Tcrδ^−/−^ mice. The results showed that the proportions of CD49f^bri^CD71^dim^ cells (Fig. 1A) and K15^+^ cells (Fig. 1B) in the normal epidermis of Tcrδ^−/−^ mice did not differ significantly from those of WT mice. Additionally, the morphology and number of K15^+^ cells also showed little or no differences between these two groups (Fig. 1C). Using a BrdU labeled mouse model, we confirmed that there were no statistically significant differences for BrdU^+^ cells in the normal epidermis between WT and Tcrδ^−/−^ mice (Fig. 1D). These findings implied that DETCs had no significant effects on the proliferation of EpSCs in the intact epidermis.

### DETCs increased the proportions of EpSCs in the epidermis around the wound area, which was beneficial for efficient wound re-epithelialization

**Fig. 2:**
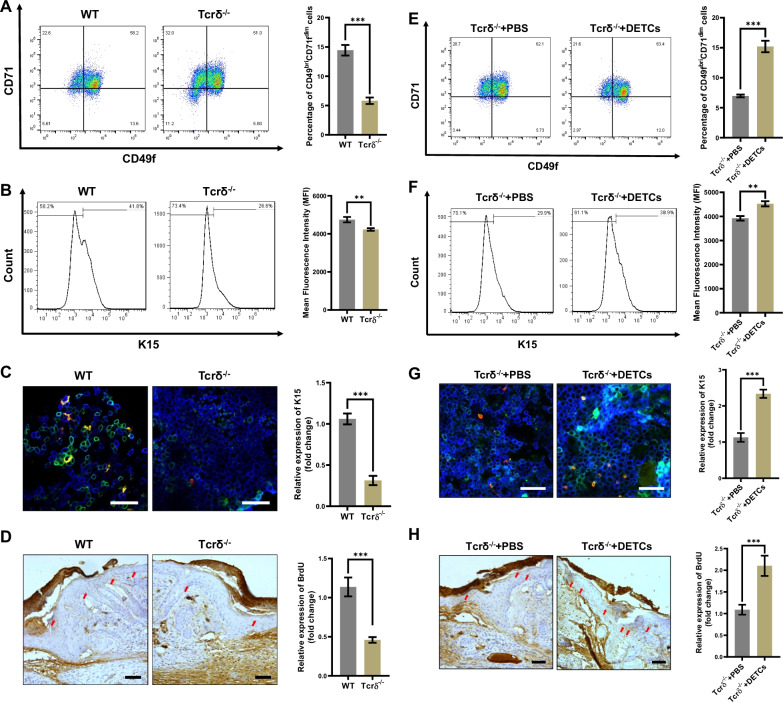
DETCs increased the proportion of EpSCs in the epidermis around the wound. **A**–**C** Full-thickness wounds were generated in age- and sex-matched WT and Tcrδ^−/−^ mice using a sterile 6-mm punch tool on day 0. Epidermal tissues around the wound were isolated on day 3 after injury. The proportions of CD49f^bri^CD71^dim^ cells (**A**) and K15^+^ cells (**B**) were detected by FACS. **C** The morphology (left panel) and number per visual field (right panel) of K15^+^ cells in the epidermis around wounds in WT and Tcrδ^−/−^ mice on day 3 after injury were analyzed by means of IF. Scale bar: 100 µm. **D** Paraffin sections of wounded skin tissues on day 3 post-wounding from 8-week-old WT and Tcrδ^−/−^ mice that had been labeled with BrdU after birth. BrdU^+^ cells in these tissues were detected by means of IHC. The arrow indicates positively stained cells (tan staining). Scale bar: 100 µm. **E**–**G** Cultured DETCs (1 × 10^5^ cells/wound) or PBS was added to the wound bed of Tcrδ^−/−^ mice. Three days later, epidermal tissues around the wound were collected. The proportions of CD49f^bri^CD71^dim^ cells (**E**) and K15^+^ cells (**F**) were detected by FACS. **G** The morphology (left panel) and number per visual field (right panel) of K15^+^ cells were analyzed by means of IF. Scale bar: 100 µm. **H** Wounded skin tissues on day 3 post-wounding from 8-week-old Tcrδ^−/−^ mice that had been labeled with BrdU as previously described were used to detect BrdU with immunohistochemical staining. The arrow indicates positively stained cells (tan staining). Scale bar: 100 µm. All data are representative of at least three independent experiments and represent mean ± SD of indicated number of mice per group. The *p* value was calculated by Student’s unpaired *t* test (**A**–**C**, **E**–**G**) (**p < 0.01, ***p < 0.001)

By promoting re-epithelialization, both DETCs and EpSCs play important roles in skin wound healing. However, the effect of DETCs on EpSCs is still unknown. To clarify the regulatory effect of DETCs on EpSCs in the process of wound healing, we collected epidermis tissues around wound areas from age- and sex-matched WT and Tcrδ^−/−^ mice on day 3 after injury. Our results showed that, compared to that of WT mice, the epidermis tissues of the skin wound area in the Tcrδ^−/−^ mice exhibited a significant decreasing proportion of CD49f^bri^CD71^dim^ cells (Fig. 2A) and K15^+^ cells (Fig. 2B). Consistently, the number of K15^+^ cells in the epidermis tissues around the wound area of Tcrδ^−/−^ mice displayed a remarkable decrease (Fig. 2C). The BrdU labeled mouse model made this effect more evident, showing that the number of BrdU^+^ cells in the epidermis tissues of the wound area from the Tcrδ^−/−^ mice notably decreased compared to that in WT mice (Fig. 2D). Furthermore, supplementation of DETCs on the skin wound bed substantially increased the populations of CD49f^bri^CD71^dim^ cells (Fig. 2E) and K15^+^ cells (Fig. 2F–G) in the epidermis tissues of skin wounds in the Tcrδ^−/−^ mice. This phenomenon was further confirmed by BrdU assays, and the results displayed that supplementation of DETCs on the skin wound bed significantly increased the number of BrdU^+^ cells (Fig. 2H) in the epidermis tissue of the skin wound area in the Tcrδ^−/−^ mice. Therefore, we confirmed that DETCs increase the proportion of EpSCs in the epidermis tissues of the wounds, which might be an important mechanism for DETCs-mediated enhancement of re-epithelialization.

### Exosomes play important roles in DETCs-mediated proliferation of EpSCs

**Fig. 3:**
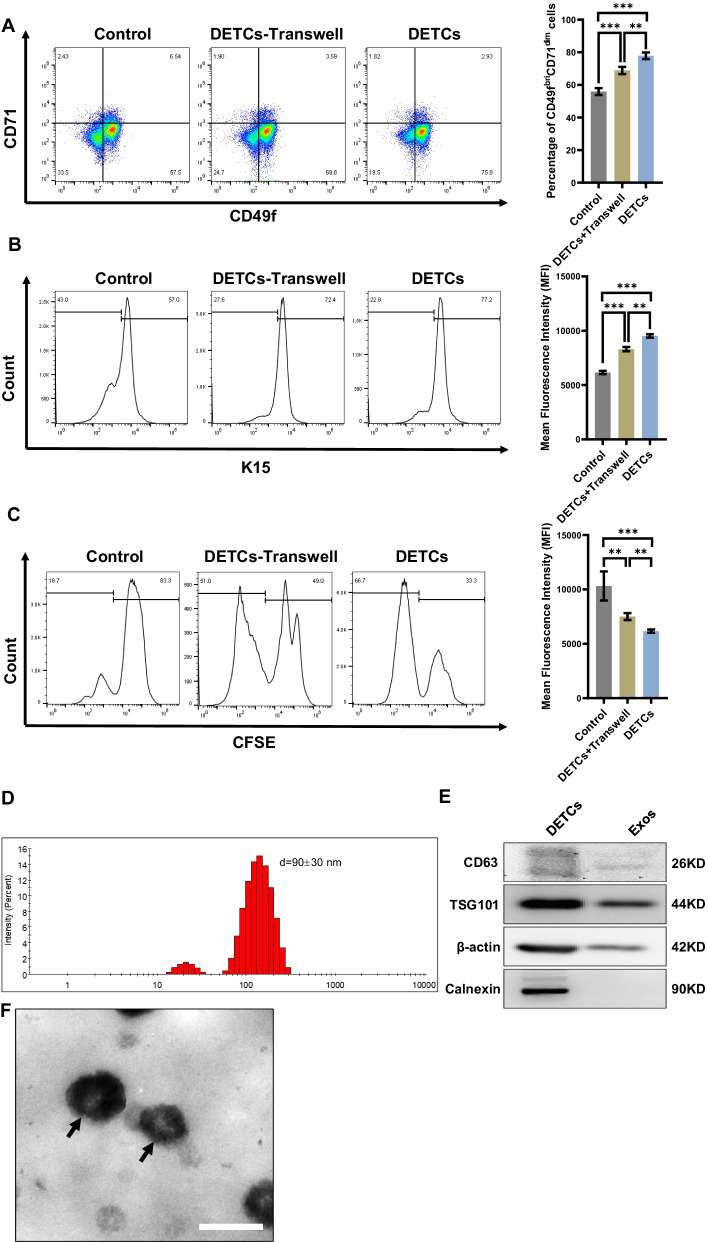
Comparative study of DETCs and EpSCs with mixed and Transwell coculture systems. **A**–**B** EpSCs were isolated from neonatal WT mice and cultured to passage 2–4 before analysis. EpSCs were cocultured with DETCs in a mixed coculture system (DETCs group) or noncontact Transwell coculture system (DETCs-Transwell group). The proportions of CD49f^bri^CD71^dim^ cells (**A**) and K15^+^ cells (**B**) were detected by FACS after 3 days of coculture. **C** EpSCs were isolated from neonatal WT mice and cultured for three days before labeling with CFSE. CFSE-labeled EpSCs were cocultured with DETCs in a mixed coculture system (DETCs group) or noncontact Transwell coculture system (DETCs-Transwell group). The proportion of CFSE^low^ cells was detected by FACS after 3 days of coculture. **D**–**F** DETCs-derived exosomes were isolated from the culture medium of DETCs. The particle size distribution of DETCs-derived exosomes was measured using a nanoparticle sizer (**D**). The expression of typical proteins (Calnexin, CD63, β-actin and TSG101) in DETCs-derived exosomes and the control group (cellular protein from 3T3 cells) was detected by means of WB (**E**). The morphology of DETCs-derived exosomes was detected by transmission electron microscopy (Black arrows indicate Exo) (**F**). All data are representative of at least three independent experiments and represent mean ± SD of indicated number of mice per group. The *p* value was calculated by one-way ANOVA with Bonferroni’s multiple comparison test (**A**–**C**) (^ns^*p* > 0.05, *p < 0.05, **p < 0.01, ***p < 0.001)

EpSCs participate in efficient skin wound healing via rapid proliferation; in other words, the proliferation of EpSCs is of great importance for wound closure [31]. Therefore, it is reasonable to assume that by promoting proliferation, DETCs increase the number of EpSCs in the epidermis of the wounds. To verify this hypothesis, we cocultured DETCs with EpSCs in a mixed coculture system (DETCs group) and a noncontact transwell coculture system (DETCs-transwell group). Compared to those in the EpSCs alone group, remarkably increased proportions of CD49f^bri^CD71^dim^ cells (Fig. 3A), K15^+^ cells (Fig. 3B) and CFSE^low^ cells (Fig. 3C) were observed in both the mixed and indirect coculture systems, suggesting that DETCs promote the proliferation of EpSCs by both contact and noncontact manners. Furthermore, the effects in the mixed group were superior than noncontact coculture groups (Fig. 3A–C). These data indicated that exosomes play an essential role in the DETCs-mediated proliferation of EpSCs.

**Fig. 4:**
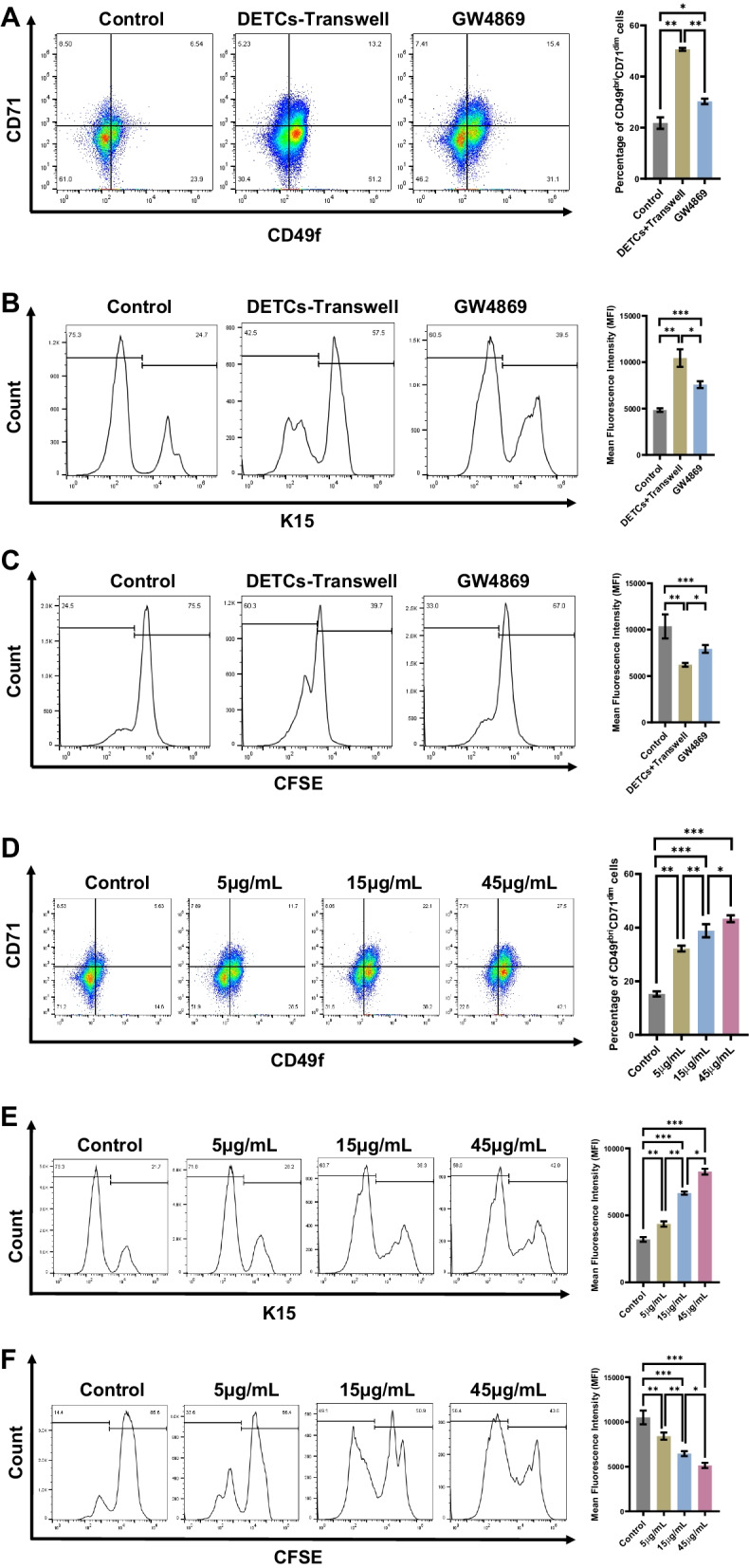
Through exosome secretion, DETCs remotely regulated the proliferation of EpSCs. **A**–**B** EpSCs were isolated from neonatal WT mice and cultured to passage 2–4 before analysis. EpSCs were cocultured with DETCs or pretreated DETCs (with GW4869) in a noncontact Transwell coculture system for three days. The proportions of CD49f^bri^CD71^dim^ cells (**A**) and K15^+^ cells (**B**) were detected by FACS after 3 days of coculture. **C** EpSCs were isolated from neonatal WT mice and cultured for three days before labeling with CFSE. CFSE-labeled EpSCs were cocultured with DETCs or pretreated DETCs (with GW4869) in a noncontact Transwell coculture system for three days. The proportion of CFSE^low^ cells was detected by FACS. **D**–**F** EpSCs were isolated from neonatal WT mice and cultured for three days before further analysis. EpSCs were cultured for three days with DETCs-derived exosomes (0 μg/mL, 5 μg/mL, 15 μg/mL, and 45 μg/mL). The proportions of CD49f^bri^CD71^dim^ cells (**D**) and K15^+^ cells (**E**) were detected by FACS. **F** EpSCs were isolated from neonatal mice and cultured for three days before labeling with CFSE. CFSE-labeled EpSCs were cultured for three days with DETCs-derived exosomes (0 μg/mL, 5 μg/mL, 15 μg/mL, and 45 μg/mL). The proportion of CFSE^low^ cells was detected by FACS. All data are representative of at least three independent experiments and represent mean ± SD of indicated number of mice per group. The *p* value was calculated by one-way ANOVA with Bonferroni’s multiple comparison test (**A**–**F**) (^ns^*p* > 0.05, *p < 0.05, **p < 0.01, ***p < 0.001)

Exosomes act as a vital messenger for mediating efficient intercellular communication among multiple types of cells [32]. To clarify the functions of DETCs-derived exosomes in DETCs-mediated regulation of EpSCs, we first isolated and identified exosomes derived from DETCs. As shown in Fig. 3D, the size of DETCs-derived exosomes was 90 ± 30 nm in diameter, as measured by a nanoparticle sizer (Fig. 3D). Moreover, unlike 3T3 cellular proteins, DETCs-derived exosomes shared a positive expression of several proteins, namely CD63, TSG101 and β-actin, but negative for Calnexin (Fig. 3E). Regarding morphology, DETCs-derived exosomes presented oval shapes with low-density areas in the middle area under a transmission electron microscopy (Fig. 3F). These findings implied that DETCs can secrete exosomes that serve as cell signaling molecules. We then further investigated whether DETCs affect the proliferation of EpSCs by producing exosomes. As expected, compared to the control group, the DETCs-Transwell group exhibited a remarkable increase for the proportions of CD49f^bri^CD71^dim^ cells (Fig. 4A), K15^+^ cells (Fig. 4B) and CFSE^low^ cells (Fig. 4C). However, when DETCs were treated with GW4869 reagent to inhibit the secretion of exosomes, the proliferation of EpSCs showed a considerable reduction (Fig. 4A–C). In line with the previous results, the addition of DETCs-derived exosomes to EpSC culture medium obviously increased the proportions of CD49f^bri^CD71^dim^ cells (Fig. 4D), K15^+^ cells (Fig. 4E) and CFSE^low^ cells (Fig. 4F). Additionally, as the concentration of DETCs-derived exosomes was increased, the proportions of CD49f^bri^CD71^dim^ cells (Fig. 4D), K15^+^ cells (Fig. 4E) and CFSE^low^ cells significantly increased (Fig. 4F). These findings revealed that DETCs promote the proliferation of EpSCs by secreting exosomes.

### DETCs-derived exosomes promote skin wound healing

**Fig. 5:**
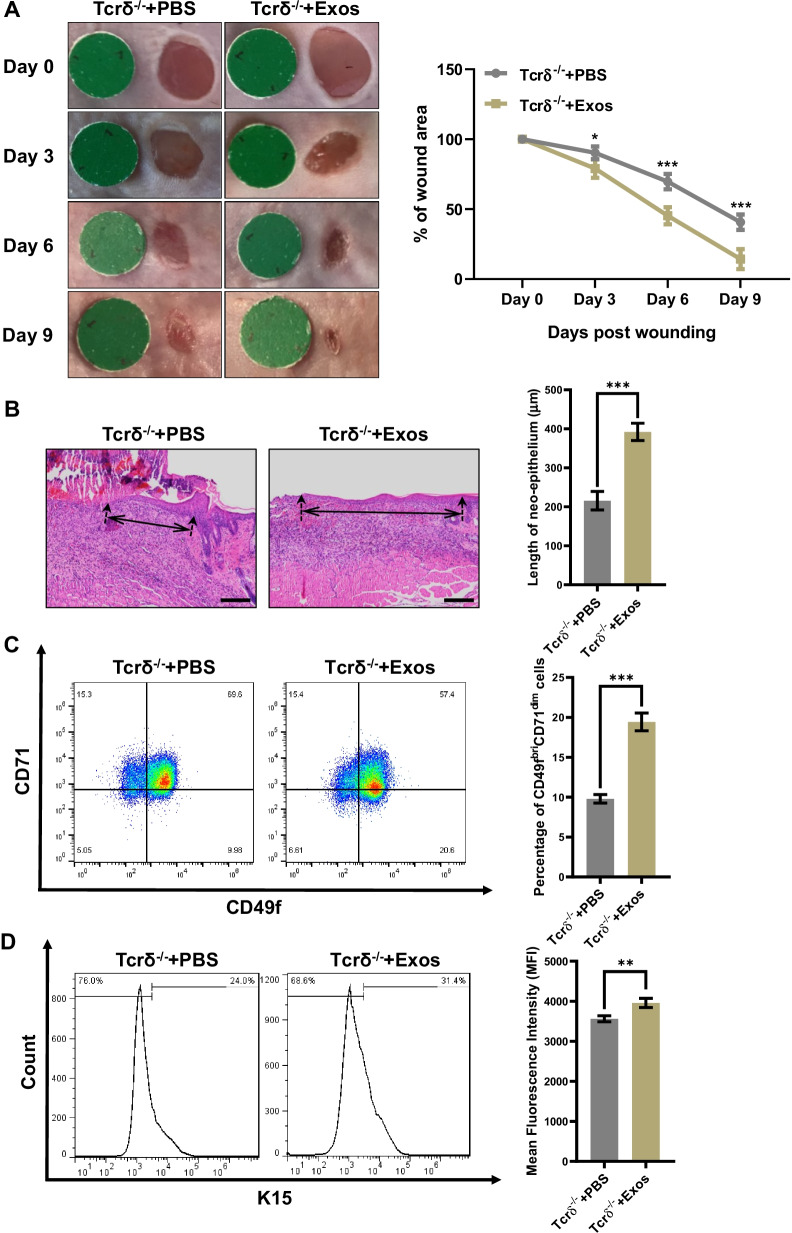
DETCs-derived exosomes promoted skin wound healing. **A**–**D** Full-thickness wounds were generated in Tcrδ^−/−^ mice on day 0, followed by application of freshly isolated Exos (15 μg/wound) or PBS onto the wound bed daily for six days after wounding. Wound closure kinetics were measured over time in the wound model without contraction (**A**). On day 3 after wound excision, re-epithelialization in the wound model without contraction (**B**) was analyzed by HE. (Black solid line with arrows indicated the neo-epithelialization length). Scale bar: 200 µm. On day 3 after wound excision, the percentages of CD49f^bri^CD71^dim^ cells (**C**) and K15^+^ cells (**D**) in the epidermis around the wounds were analyzed by FACS. All data are representative of at least three independent experiments and represent mean ± SD of indicated number of mice per group. The values were calculated as the mean ± SD. The *p* value was calculated by Student’s unpaired *t* test (**A**–**D**) (*p < 0.05, **p < 0.01, ***p < 0.001)

As mentioned above, it has been demonstrated that DETCs-derived exosomes promoted the proliferation of EpSCs in vitro. We continued to determine the effects of DETCs-derived exosomes on the wound healing in vivo. According to the results, after treated with DETCs-derived exosomes on the skin wound bed, the Tcrδ^−/−^ mice displayed notably improved wound healing on day 3, 6 and 9 (Fig. 5A) and re-epithelialization on day 6 post-wounding (Fig. 5B). Furthermore, we collected epidermis tissues from skin wounds of the control group (Tcrδ^−/−^ mice + PBS) and experimental group (Tcrδ^−/−^ mice + DETCs-derived exosomes) 3 days after injury. It was observed that the Tcrδ^−/−^ mice exposed to DETCs-derived exosome displayed significantly higher proportions of CD49f^bri^CD71^dim^ cells (Fig. 5C) and K15^+^ cells (Fig. 5D) in the epidermis tissues of the skin wounds. Overall, we concluded that DETCs remotely regulate the proliferation of EpSCs via the secretion of exosomes to promote cutaneous wound healing.

## Discussion

DETCs, a major subset of T cells in the epidermis tissues of skin, play important roles for efficient skin wound healing [33]. By providing a major source of growth factors, such as IGF-1 and KGF, in the epidermis tissues around the wounds, DETCs promote re-epithelialization to accelerate cutaneous wound repair [34]. The migration, proliferation, and differentiation of EpSCs play crucial roles in the process of re-epithelialization [7]. However, how DETCs regulate the cellular behavior of EpSCs to enhance re-epithelialization is still unclear. Herein, we demonstrate for the first time that skin-resident DETCs promote the proliferation of EpSCs to enhance re-epithelialization, in which DETCs-derived exosomes are the vital effector.

By promoting the proliferation, differentiation and migration of keratinocytes and protecting them against apoptosis, DETCs enhance the re-epithelialization for facilitating skin wound closure [35]. Keyes BE et al. have documented that the Tcrδ^−/−^ mice exhibit a weakened re-epithelialization and delayed wound healing, suggesting that DETCs, to a large extent, contribute to skin regeneration [36]. The proliferation of EpSCs is required for maintaining sustained proliferation and differentiation of keratinocytes, so maintaining an appropriate proportion of EpSCs in the epidermis of wounds is essential for proper wound re-epithelialization and healing [31]. To investigate the effects of DETCs on the proliferation of EpSCs in vitro and their roles in promoting the re-epithelialization in vivo, we applied three biomarker systems (CD49f-CD71, K15, and BrdU) to identify the variation of EpSCs. These data uncovered that DETCs deficiency profoundly reduced the population of EpSCs in the wounded epidermis, and this effect was rescued by the addition of DETCs into the wounds, suggesting that DETCs might increase the recruitment and/or proliferation of EpSCs in the epidermis around the wounds to accelerate the re-epithelialization. Therefore, we further detected the effects of DETCs on the proliferation of EpSCs using a CFSE labeling assay by a coculture system of EpSCs and DETCs in vitro, disclosing that DETCs markedly promoted the proliferation of EpSCs. Taken together, these data indicate that the enhancement of EpSCs proliferation can be an important regulatory mechanism by which DETCs promote re-epithelialization.

DETCs keep the homeostasis of cutaneous epidermal tissues and promote wound re-epithelialization through the paracrine mode of a series of growth factor [37]. Furthermore, using a mixed coculture system (including direct and indirect intercellular communication) and a noncontact transwell coculture system (including indirect intercellular communication only), our data have implied that, as a remote regulatory mechanism, DETCs increase the proliferation rate of EpSCs via a noncontact manner. It has been well-known that DETCs primarily provide the source of IGF-1 and KGF-1/2 in the epidermis to enhance re-epithelialization and thereby promote skin wound repair [19]. IGF-1 and KGF-1/2 play critical roles in DETCs-mediated indirect regulation of the migration, proliferation and morphogenesis of keratinocytes. In addition, exosomes, a highly effective manner of intercellular communication, are frequently used to transduce signals and messages by many types of immune cells involving B lymphocytes, dendritic cells, and mast cells, which play a leading role in regulating the proliferation and differentiation of different types of stem cells (e.g., tumor stem cells, mesenchymal stem cells, and epidermal progenitor cells) [38–41]. However, the role of exosomes in the regulation of EpSCs proliferation by DETCs is still vague. In the current study, a large number of exosomes were isolated from the culture medium of DETCs and confirmed by morphological examination, diameter evaluation, and typical biomarker identification. It is revealed the crucial roles of exosomes in the DETCs-mediated enhancement of EpSCs proliferation. In the transwell coculture system of EpSCs and DETCs, blocking exosome secretion in DETCs nearly eliminated the effects of DETCs on the proliferation of EpSCs. Similarly, EpSCs exposed to DETCs-derived exosomes nearly replicated the effects of DETCs on the proliferation of EpSCs. More importantly, the application of DETCs-derived exosomes into the wound bed partially replicated the phenomena associated with DETCs administration, for example, an increased proportion of EpSCs in the epidermis of wounds, the enhanced wound re-epithelialization (data not shown), and the increased cutaneous wound closure (data not shown). These data strongly demonstrated that exosomes derived from DETCs promoted the proliferation of EpSCs for accelerating the wound healing.

As a skin stem cell, EpSCs have the potential to differentiate into multiple cell lines that replenish wounded area after cutaneous injury [42]. For tissue regeneration and repair, different therapy strategies including adipose-derived mesenchymal stem cells (AD-MSCs), stromal vascular fraction (SVF), and exosomes combined with dermal substitute and autologous growth factors like to platelet-rich plasma (PRP) have been employed for clinical application [43–46]. On the one hand, Zhao et al. [43] reported that eighteen patients with knee osteoarthritis were performed using allogenic AD-MSCs in a randomized clinical trial for cartilage repair. A similar study found by Valerio et al. [44] uncovered that SVF and PRP mixed with fat grafting showed significant improvement compared with either SVF or PRP treatment in these patients suffered post-traumatic extremity ulcers. On the other hand, biomaterials-based exosome delivery systems were used for organization in various forms, including nanofiber meshes, hydrogels, porous scaffolds, and decellularized matrices [46]. These biomaterials contribute to maintain exosome release by passive strategies such as incubating exosomes with the appropriate polymers or active strategies including electroporation, extruding, and click strategy to repair damaged tissues [45]. However, more underlying molecular mechanisms and preclinical trial schemes further need to be investigated and carried out before extensive clinical application.

There are several points from this article that need to be clarified. First, we found that deficiency of DETCs in the epidermis had no obvious effects on the maintenance of the EpSCs population in the normal epidermis. One cause may be the inactivity and slow-cycling property of EpSCs and DETCs, when the skin is intact. Second, there are different methods of EpSCs identification, and different studies may use different biomarkers. To avoid incomplete information arising from one-sided identification of EpSCs, three biomarker systems were used in our research. While there were marked differences between the three biomarker systems in identifying EpSCs, the overall trend was identical, which indicated that our conclusions were credible. Third, as an extracellular vesicle released by cells, exosomes contain various kinds of proteins, DNAs and RNAs. Further studies need to be performed to understand which contents of exosomes facilitate the proliferation of EpSCs. Our data highlight exosome as a crucial signaling transducer employed by skin-resident DETCs to facilitate the wound re-epithelialization by promoting the proliferation of EpSCs in the wound bed. Furthermore, our research provides novel insights into the complex interrelationship between skin-resident immune cells and EpSCs during wound repair, indicating that DETCs-derived exosomes might be exploited in the stem cell-based regenerative medicine.

## Conclusion

In this study, we have demonstrated that DETCs increased the proportion of EpSCs in the epidermis tissues of wounds by exosomes to accelerate re-epithelialization. Taken together, our data clarified a novel mechanism among DETCs-derived exosomes, EpSCs, and wound re-epithelialization in the wounded skin, which might provide a new perspective for clinical wound therapy and prognosis.

## Data Availability

The data and material support that all findings could be found.

## Abbreviations

AD-MSCs: Adipose-derived mesenchymal stem cells
BrdU: Bromodeoxyuridine
CFSE: Carboxyfluorescein diacetate succinimidyl ester
DETCs: Dendritic epidermal T cells
EpSCs: Epidermal stem cells
Exo: DETCs-derived exosome
FBS: Fetal bovine serum
IGF: Insulin-like growth factor
KGF: Keratinocyte growth factor
Nrf2-ADSC-Exos: Exosomes derived from adipose stem cells overexpression nuclear factor-E2-related factor 2
PRP: Platelet-rich plasma
PVDF: Polyvinylidene difluoride
SVF: Stromal vascular fraction
Tcrδ-/-: Tcrδ knockout
TEM: Transmission electron microscopy
WT: Wide type
